# Medical News

**Published:** 1852-05

**Authors:** 


### MEDICAL NEWS.

Death of Professor Grant.—At a meeting of the medical Faculty of Pennsylvania College, held March 29th, 1852, the following preamble and resolutions were unanimously adopted:

*Whereas,* It has pleased the Supreme Disposer of events to remove from our midst, our beloved friend and colleague, Dr. William R. Grant, Professor of Anatomy in this school since 1843 : therefore

*Resolved,* That this Faculty is deeply sensible of the loss it has sustained in the decease of one so long endeared to its members, by his purity of heart, his amiability of character, his uniform tenderness and courtesy, his professional zeal and ability, and his devotion to the duties of the chair of which he was the incumbent.

*Resolved,* That, in token of respect to the memory of Professor Grant, the Faculty will attend his funeral in a body; and that one of its members be appointed to prepare a biographical sketch of the deceased, to be delivered as an introductory lecture to the session of 1852–53.

D. Gilbert M. D., *Registrar.*

Naval Surgeons.—The Board of Naval Surgeons, recently convened in this city for the examination of Assistant Surgeons, have reported the following duly qualified for promotion, viz: Washington Sherman, of the date of 1845, to take rank next after Passed Assistant Surgeon William A. Harris. Randolph F. Mason, of the date of 1846, take rank next after Passed Assistant Surgeon Henry O. Mayo; and John Rudenstein, of the date of 1846, to take rank next after Passed Assistant Surgeon Randolph F. Mason. The Assistant Surgeons of the date of 1847, who have passed, to take effect from the 26th of April 1852, will rank in the following order, viz.: Edward R. Squibb, Robert J. Farquharson, Philip Lansdale, James F. Harrison, James S. Gilliam, J. W. B. Greenhow.

Relative Mortality of the States.—We have seen it stated that the proportion of deaths to the population in the several States of the Union, as shown by the census of 1850, is as follows: Vermont, 1 in 100; Iowa, 1 in 94; Georgia, 1 in 91; Michigan, 1 in 87; Tennessee, 1 in 86; North Carolina and Alabama, 1 in 85; South Carolina, 1 in 83; Maine, 1 in 77; New Jersey, 1 in 75; Virginia, 1 in 76; Illinois and Delaware, 1 in 73; Arkansas, 1 in 70; Texas, 1 in 69; Rhode Island, 1 in 56; Kentucky and Connecticut, 1 in 64; Maryland, 1 in 60; and Massachusetts 1 in 51.

